# Mind the Gap: LRRK2 Phenotypes in the Clinic vs. in Patient Cells

**DOI:** 10.3390/cells10050981

**Published:** 2021-04-22

**Authors:** Liesel Goveas, Eugénie Mutez, Marie-Christine Chartier-Harlin, Jean-Marc Taymans

**Affiliations:** 1 UMR-S 1172—LilNCog—Lille Neuroscience & Cognition, Université de Lille, Inserm, CHU Lille, F-59000 Lille, France; liesel-mary.goveas@inserm.fr (L.G.); eugenie.mutez@chru-lille.fr (E.M.); 2 Neurology and Movement Disorders Department, CHU Lille University Hospital, F-59000 Lille, France

**Keywords:** LRRK2, Parkinson’s disease, phenotypes, neurodegenerative disease, physiopathology, pathogenic mutants

## Abstract

Mutations in the Parkinson’s disease (PD) protein Leucine Rich Repeat Kinase 2 (LRRK2) have been under study for more than 15 years and our understanding of the cellular phenotypes for the pathogenic mutant forms of LRRK2 has significantly advanced. In parallel to research on LRRK2 mutations in experimental systems, clinical characterization of patients carrying LRRK2 mutations has advanced, as has the analysis of cells that are derived from these patients, including fibroblasts, blood-derived cells, or cells rendered pluripotent. Under the hypothesis that patient clinical phenotypes are a consequence of a cascade of underlying molecular mechanisms gone astray, we currently have a unique opportunity to compare findings from patients and patient-derived cells to ask the question of whether the clinical phenotype of LRRK2 Parkinson’s disease and cellular phenotypes of LRRK2 patient-derived cells may be mutually informative. In this review, we aim to summarize the available information on phenotypes of LRRK2 mutations in the clinic, in patient-derived cells, and in experimental models in order to better understand the relationship between the three at the molecular and cellular levels and identify trends and gaps in correlating the data.

## 1. Introduction

A systematic study published by the “The Global Burden of Diseases, Injuries, and Risk Factors” team in 2018 indicated that 6.1 million individuals were affected by Parkinson’s disease (PD) globally in the year 2016, which is more than double the number of affected individuals as of 1990 [1]. The International Parkinson and Movement Disorder Society recognizes PD on similar lines as that described by Hoehn and Yahr in 1967 [2]—a neurological disorder with four cardinal symptoms, namely “bradykinesia, rigidity, asymmetric resting tremor and postural instability”, although, it is not necessary for all the symptoms to manifest for a patient to be diagnosed with PD. The patients may also present with non-motor symptoms such as autonomic dysfunction (such as orthostatic hypotension, urinary and bowel disturbances), neurobehavioral abnormalities, sensory abnormalities (paresthesia’s and pain), depression, sleep disturbances (rapid eye movement sleep behavior disorder (RBD)), and cognitive decline. Clinically, PD patients also present with secondary motor symptoms like dysarthria, swallowing disorders, freezing of gait, dystonia, festination, and falls [3,4]. The treatment is only symptomatic and is mainly based on dopamine replacement therapy with levodopa. At later stages of the disease, when motor fluctuations and levodopa-induced dyskinesia appear, an alternative symptomatic therapy involves the placement of a deep brain stimulation device to re-equilibrate basal ganglia thalamocortical circuitry [5]. Neuropathologically, PD is characterized by the loss of dopaminergic (DA) neurons in the *Substantia nigra pars compacta* (SNpc) as well as by the presence of α-synuclein rich deposits in surviving neurons called Lewy bodies (LB). LBs were first discovered in the SNpc in PD in 1912; this neuropathologic finding is common to dementia with Lewy bodies (DLB), idiopathic PD (IPD), and the dementia seen in PD patients (Parkinson’s Disease Dementia [PDD]) [6]. Histopathological studies by Ishizawa and colleagues [7] revealed that there is an abnormal accumulation of phosphorylated microtubule-associated protein tau in the LBs of PD patients.

Research in the etiology of PD has revealed several risk factors that can be broadly categorized under age, environmental factors, and genetic factors [8]. These latter risk factors have been revealed through genetic linkage and genome-wide association studies (GWAS). The linkage studies are performed in families where it is suspected that there is an inherited form of PD. By applying Mendelian principles of inheritance, a gene locus can be identified (for loci linked to PD transmission, these have been dubbed PARK loci) and further sequencing or gene copy number of this locus leads to the identification of the genetic variation underlying the inheritance (such as a point mutation or gene multiplication). GWAS studies in PD are complementary to the linkage analyses and are based on the identification of single nucleotide polymorphisms (SNPs) that are enriched in a population of sporadic PD patients compared to a matched control population. Interestingly, results from both the genetic linkage and GWAS studies reveal that the leucine-rich repeat kinase 2 gene (*LRRK2*) is one of the key genetic determinants of PD. Indeed, linkage studies show mutations in the *LRRK2* coding sequence significantly segregating with PD. While familial forms of PD are not the majority of PD cases, it is of interest that mutations in *LRRK2* are among the most common causes of familial PD [9]. Furthermore, GWAS studies show several SNPs enriched in the PD population at the *LRRK2* locus [10]. While disease risk of coding mutations in *LRRK2* is much higher than for the SNPs at the *LRRK2* locus, the identification of *LRRK2* as a risk factor for PD in both linkage and association studies indicates that *LRRK2* is relevant to disease in both familial and sporadic forms of PD.

**Figure 1:**
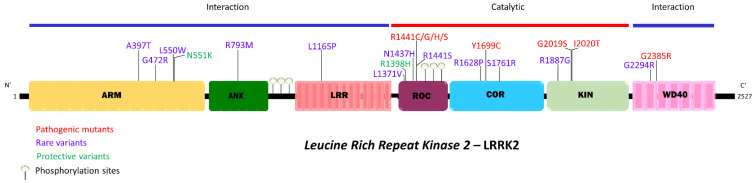
Diagrammatic representation of Leucine Rich repeat Kinase 2 (LRRK2). Depicted are the functional domains of LRRK2. Different variants are indicated in different colors, including pathogenic variants (red), rare variants potentially pathogenic (purple), and protective variants (green). ARM, armadillo repeat domain; ANK, ankyrin repeat domain; LRR, leucine-rich repeat domain; ROC, Ras of complex proteins domain; COR, C-terminal of ROC domain; KIN, kinase domain; WD40, WD40 repeat domain.

LRRK2 protein is a large multidomain protein, with an evolutionarily conserved Ras of complex (ROC) GTPase connected via a C terminal of ROC (COR) to a kinase domain, and thus belongs to a family of GTPases with a characteristic tandem of ROC-Cor domain and is referred to as a ROCO protein [11,12,13]. Besides the catalytic domains, LRRK2 comprises protein–protein interaction domains, including in the N-terminal Armadillo domain (ARM), an Ankyrin domain (ANK), and Leucine-rich repeat domain (LRR) and in the C-terminus, a WD40 domain (Figure 1 adapted from the paper by *Civiero* and colleagues [14]). Strikingly, LRRK2 is phosphorylated at multiple sites, with a cluster of heterologous phosphorylation sites in the ANK-LRR interdomain region and an autophosphorylation cluster in the ROC domain [15].

Research on the functions of LRRK2 in the past 15 years have begun to paint a picture of what the normal functions of LRRK2 are. The GTPase function of LRRK2, which lies in the catalytic core of LRRK2, is known to functionally interact with the kinase domain; further, the autophosphorylation potential of LRRK2 is also known to increase its GTPase activity [16]. The R1441C/G/H and Y1699C variants lead to a significant decrease in GTPase activity, whereas the G2019S and I2020T mutants display unchanged GTPase activity (reviewed by Nguyen and colleagues [17]). The kinase activity of LRRK2 is reflected by its capacity to autophosphorylate as well as to phosphorylate physiological substrates, including several Rab GTPase proteins [18,19]. While observations show some differences in kinase activity between mutants, overall, it can be stated that the majority of pathogenic mutants lead to increased kinase activity relative to the WT protein, with some exceptions. For instance, for the R1441C mutant, a minority of studies report slightly decreased kinase activity [20,21]; whereas, for the G2385R mutant, the majority of studies report a decreased kinase activity. Dimerization is a common phenomenon observed among kinase proteins but is not a characteristic of ROCO proteins. Several domains of LRRK2 (ROC-COR, WD40), and not only the kinase domain, are involved forming a stable dimer and promoting autophosphorylation [22,23,24].

Cell biological studies show that LRRK2 plays a normal role in neurite outgrowth, mitochondrial functions, cytoskeletal maintenance, vesicle trafficking, regulation of the endolysosomal system, autophagic protein degradation, the immune system, or protein translation [25,26,27]. Although the precise biological role of LRRK2 is yet to be defined, perhaps the best clues come from network analysis studies and studies with LRRK2 Knockout (KO) animals. Indeed, LRRK2 interactomic studies assign to LRRK2 a role in intracellular transport and organization [28,29]. This is consistent with findings from morphological and histopathological examination in homozygous LRRK2KO rats showing that there is a progressive age -related abnormality that is observed in the kidney, lungs, and liver, notably in the form of increased vacuolation [30]. The studies in homozygous LRRK2 KO mouse models also show age dependent increased renal atrophy and degeneration, an abnormal accumulation of α-synuclein and ubiquitinated protein, an impairment of the autophagy-lysosomal pathway, but no neurodegeneration [31,32,33]. Other studies in LRRK2 KO animals suggest that LRRK2 is a modulator of toxic insults, including a modulator of experimental colitis [34], susceptibility to carcinogen-induced lung cancer [35], and sensitivity to alpha-synuclein mediated nigrostriatal neurodegeneration [36].

## 2. Understanding LRRK2 in PD through the Study of Disease-Linked or Associated Variants of LRRK2

The LRRK2 mutations that impact disease by increasing or decreasing the risk of PD are of utmost importance (see schematic overview in Figure 1). Based on this, we can discern different types of mutations in LRRK2. Several mutations have been demonstrated via the linkage studies to significantly segregate with the disease, including N1437H, R1441C/G/H, Y1699C, G2019S, I2020T, and G2385R. Interestingly, these confirmed disease-linked mutations in LRRK2 are mostly found in the catalytic core of LRRK2. Other mutations are hypothesized to confer risk for disease. However, their pathogenic nature could not (yet) be confirmed as they do not occur frequently enough to perform an adequately powered statistical test. Such mutations are labeled here as ‘rare variants’ and include A397T, G472R, L550W, R793M, L1165P, R1441S, S1761R, and D1887G. Finally, other variants do not confer risk for disease, but rather are inversely correlated to disease risk and are therefore described as protective. Examples of protective mutations are N551K and R1398H.

For the sake of being complete, we should mention the SNP variants identified in GWAS studies. Many SNPs at the *LRRK2* locus associated with PD have not been shown to alter the LRRK2 protein sequence, although there are examples of low-risk mutations in the coding sequence, and effects on splicing or in noncoding sequences regulating expression level cannot be excluded. Significantly associated genome-wide PD SNPs at the *LRRK2* gene are: rs11175620, rs76904798, rs7294619, rs10878226, rs1491942, rs11175645, rs11175655, rs11175658, rs74324737, rs11175666, rs34637584 [37], rs28903073 [38], rs190807041 [39], and rs1491923 [40]. In addition, some risk factor SNPs that introduce an amino-acid substitution in the LRRK2 protein sequence are rs34778348 (G2385R, c.7153G>A) [41], rs33949390 (R1628P, c.4883G>C) [38], and rs11564148 (S1647T) [42].

The identification of the precise genetic variants in the *LRRK2* gene or at the *LRRK2* locus that are implicated in PD is a very valuable starting point for studies researching the pathological role of each variant. A first approach consists of studies in experimental systems to identify the biochemical, cell biological, and systems-level effects of each variant. This approach involves purifying LRRK2 protein of the different mutants and carrying out a biochemical characterization by performing enzymatic or dimerization tests and expressing LRRK2 mutants in cell lines or in animal models to test effects on readouts such as viability or cellular functions such as cytoskeleton regulation or membrane trafficking. Of course, particular attention can be given to the biochemical function mediated by the specific domain where the mutation is found, such as GTPase activity for mutants in the ROC-GTPase domain or kinase activity for mutations in the kinase domain. Parallel to this, clinicians can establish a detailed profile of clinical symptoms associated with each mutation. Clinical phenotypes in the field of PD can be characterized by the presence of motor symptoms that that may or may not manifest along with non-motor symptoms and it is valuable to know whether these LRRK2 variants give rise to a clinical phenotype close to or indistinguishable from the phenotype exhibited by idiopathic PD patients. Another approach is to study the effects of LRRK2 variants in cells cultured from PD patients who carry LRRK2 variants and establish an in-depth characterization of the patient-derived cells. This approach is rather attractive as the cells analyzed harbor not only the PD associated LRRK2 variant, but also potential other elements in the genetic background that may contribute to increasing or decreasing disease risk. The flexibility of this approach is illustrated by the variety of cell types that can be implemented. These include primary cultures such as fibroblasts from skin biopsies or cultured peripheral blood mononuclear cells (PBMC), as well as modified cells such as lymphoblastoid cell lines derived from EBV immortalized lymphocytes, induced pluripotent stem cells (iPSCs) obtained by dedifferentiation of primary cells, and the use of the iPSCs to obtain redifferentiated populations of cells, for instance to neuronal, glial (information on row 17, Table 1), or other cell types.

**Figure 2:**
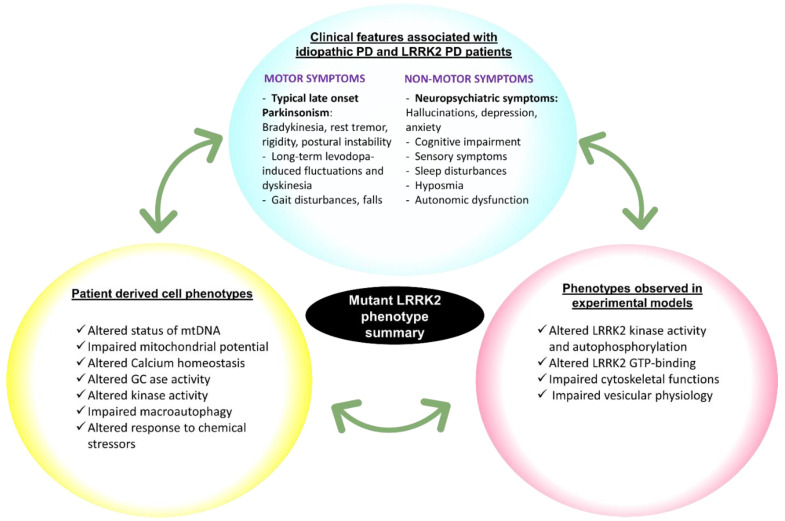
Diagrammatic representation of the summary of the mutant LRRK2 phenotypes observed in PD patients, patient-derived cells, and in experimental models. The diagram first summarizes the present review to describe the phenotypes of mutant LRRK2 in the three areas (given as three bubbles) of the clinic, patient-derived cells, and experimental models. Each bubble highlights the main phenotypic observations for that area. Please refer to the text for a detailed mutant per mutant description of the different phenotypes. Please also refer to Table 1 for a detailed inventory of the patient-derived cell phenotypes described in literature.

Studies describing clinical phenotypes of patients carrying LRRK2 variants as well as work on cells cultured from these patients have emerged in recent years and makes feasible a search for correlations between the patient clinical symptoms, patient-derived cell phenotypes, and the cellular and molecular effects of LRRK2 variants. Therefore, the aim of this review is to provide an overview (summarized in Figure 2) of (i) what we currently know of the patient characteristics and (ii) patient-derived cell phenotypes for each LRRK2 variant as well as (iii) a brief reminder of observed dysfunctions of LRRK2 variants from studies in experimental systems. For this latter point, please note that this is developed in more details in several recent reviews, including references [43,44,45]. It should also be noted that for clinical features of mutation carriers, a distinction can be made between studies that offer a description of specific cases and studies that have analyzed groups of mutation carriers. The overview of the literature on each LRRK2 mutation given below is organized domain per domain.

### 2.1. ARM—ANK—LRR Domains

#### ARM—ANK—LRR Clinical Characteristics

**A397T**: Kishore and colleagues [46] identified the A397T mutation in two female [F] PD patients of Indian descent who developed PD in their mid-fifties, with a disease duration of nine (A) and sixteen years (B). Both patients presented with rigidity and bradykinesia as motor symptoms, patient A also displayed cognitive dysfunction, whereas patient B displayed urinary urgency, constipation, anxiety, and depression as non-motor phenotypes. Both the patients’ treatment strategies involved surgical intervention (right pallidotomy (patient A), bilateral *subthalamic nucleus*—Deep Brain Stimulation (STN DBS) (patient B)), to which they responded well [46].

**G472R**: Another reported described a female PD patient with a G472R with an onset age of fifty-six years, disease duration of fifteen years, and presenting with tremor, bradykinesia, and rigidity as motor symptoms. Non-motor symptoms include REM sleep Behavior Disorder (RBD), constipation, urinary urgency, anxiety, and depression. The patient responded well to treatment by bilateral STN DBS [46].

**L550W**: Three patients are reported in the Indian population reporting to carry the L550W mutation, patient A (F), patient B (Male (M)), patient C (F), with age of onset at 47, 45, and 58, respectively. Patients presented with rigidity (A, B, C), bradykinesia (A, B, C) and tremor (B). Patients B complained of constipation, urinary urgency, and excessive daytime somnolence, whereas patient C complained about insomnia. Levodopa was determined as the best therapeutic strategy for the patients [46].

**N551K**: The mutation was reported in [10]. However, as no detailed information is available, further studies are required to determine the clinical features of this mutation.

**R793M**: This mutation was reported first by Covy and colleagues [47], and the patient was responsive to dopamine replacement therapy, (age of diagnosis 77) but developed progressive disability over a period of 15 years (died at 92). Histopathological examination of brain tissue revealed depigmentation of the SNpc and *locus coeruleus (LC)*, abundant Lewy bodies (LB), Lewy neurites (LN), and α-syn immuno-reactive spheroids in the SNpc. A moderate number of Neurofibrillary tangles (NFTs) and tau-positive dystrophic neurites were observed in the hippocampal formation, senile plaques (SP) were rare, and no LRRK2 inclusions were observed.

**L1165P**: The L1165P mutation reported in a male PD patient case indicating an early onset PD (onset at 47), characterized by presence of cardinal symptoms, without sensory impairments, responsive to carbidopa/levodopa, auditory/visual hallucinations in response to the dopamine replacement therapy developed within 10 years of disease onset. Non-motor symptoms gradually deteriorated the patient’s condition followed by death at age 81. Histopathological studies revealed loss of pigment in the SNpc/LC, abundant presence of LBs (SNpc), LNs (SNpc), α-syn immuno-reactive spheroids (SNpc, amygdala, hippocampal formation, sparse in neocortex), and rare tau-inclusions and SPs [47].

As of the writing of this review, few studies have been published characterizing the effects of these mutations on LRRK2 biology. In addition, at present, no patient-derived cells from carriers of these mutations have been reported.

### 2.2. ROC Domain

Disease-related mutations in the ROC domain include the pathogenic mutations N1437H, R1441C/G/H, the rare variant R1441S, as well as the protective variant R1398H.

#### 2.2.1. ROC—Clinical Characteristics

**R1398H**: The R1398H has been shown to be protective against PD. The mutant was enriched in the cohort of Han Chinese population and is associated with a decreased risk of PD in this population [48].

**N1437H**: A potentially pathogenic mutation in the Roc-GTPase domain of LRRK2 is described in detail by the author. The p.N1437H mutation carrier had a 19-year disease progression and displayed levodopa responsive PD, tremors, severe motor fluctuations, painful ON-dystonia, severe depression accompanied by suicidal thoughts, reduction in cognition during motor OFF, and no visible cognitive decline. Bilateral deep brain stimulation had no effect on the patient. Neuropathological examination of the brain revealed cell loss and α-synuclein positive Lewy pathology in the brain stem. However, Lewy pathology was only sparsely distributed in the cortex. No autonomic nervous system dysfunction was reported, and a pronounced ubiquitin-positive pathology was observed in brainstem, neocortex, temporolimbic region, and white matter. This pattern of ubiquitinated protein pathology is unique to this particular study and has never been reported to be so profound in other studies of brain tissue with LRRK2 mutations [49].

**R1441C**: The first detailed description of the R1441C mutation carriers was given by Haugarvoll and colleagues [50]. The paper describes that patients display a textbook representation of cardinal symptoms with a range of non-motor symptoms (hallucinations, depression, and anxiety), typical of late-onset sporadic levodopa-responsive Parkinsonism. In a cumulative study, it was noted that the R1441C mutant carriers developed symptoms before the ages of 50 (less than 20% of representative population) and 75 (>90%). Of note, the authors have noted that there might be an over-estimation of the penetrance from these numbers as the study focuses on multi-case PD families. More often, R1441C carriers may be diagnosed with Lewy Body dementia (LBD) with DA cell loss in the SNpc, without tau pathology.

**R1441G**: The R1441G mutation was initially reported in Basque families with a 100% penetrance. Simon-Sanchez and colleagues reported that this mutation accounts for about 8% of the sporadic and 20% of the familial PD cases in Basque populations [51]. Another study in 2010 by Ruiz-Martínez J and colleagues reported that the R1441G mutation accounted for 46% of familial PD and 2.5% of sporadic PD patients in the Basque population. The same study reported a lifetime penetrance of this mutation between 12.5% at the age of 65 and 83.4% at the age of 80 [52].

R1441G carriers display a mild form of disease, which is responsive to dopamine replacement therapies, levodopa, and a lack of cognitive deficits even with prolonged periods (up to 21 years) of disease progression. Of interest, unilateral tremor is a feature common to almost all R1441G carriers as reported by [51]. Hatano and colleagues reported R1441G mutant carrier patients in the Asian population and the patients showed fewer of the non-motor features as seen in IPD patients, no autonomic dysfunctions that included bowel and urinary dysfunctions and orthostatic hypotension, REM sleep behavior disorders, and reduced cardiac ^123^I-MIBG (diagnostic imaging agent) uptake [53]. A new study published in medrxiv from Fan and colleagues shows an increase of LRRK2 kinase activity-dependent Rab10-Thr73 phosphorylation in PD R1441G patient-derived peripheral blood neutrophils by over two fold [54]

**R1441H**: Ferreira JJ and colleagues described the clinical characteristics of two PD patients R1441H (one male (M) and one (F) with this heterozygous mutation in a family. The symptoms developed at 32 years (M) and 57 years (F), respectively (disease duration during study—24 years). The patient’s symptoms began with rigidity and bradykinesia initially and on examination for this particular study, the authors report no visible cognitive deficits. Both were undergoing levodopa therapy post the confirmation of the PD diagnosis. Peak-dose dyskinesia’s and visible motor fluctuations were observed in one patient 8 years post treatment with levodopa, while the other patient developed these complications between the 10th and 19th year of therapy. The female patient also additionally presented with insomnia, bladder dysfunction accompanied with sensory complaints, whereas the male patient presented with constipation, sensory complaints, and hallucination with vivid dream patterns [55].

**R1441S**: The R1441S mutation constitutes the fourth mutation possible on the R1441 residue, making this residue a mutational hotspot. The mutation was first described in detail by Mata and colleagues. Two patients were thoroughly assessed and the authors reported that the symptoms included resting tremor rigidity, bradykinesia, and unilateral onset with PD responsive to Levodopa treatment. The age range varied between 41 and 76 years, and patients suffered from mild cognitive deficits slightly prior to the onset of motor symptoms [56].

#### 2.2.2. ROC—Patient-Derived Cell Phenotypes (See Table 1)

Table 1 lists the studies with patient-derived cells including primary cells such as fibroblasts, Lymphoblastoid cells (LCLs), PBMCs as well as or iPSCs that themselves have either been tested in their pluripotent state or have been reprogrammed into a multitude of neural cell types like neural precursor cells, DA neurons, astrocytes, sensory and glutaminergic neurons, and neuro-epithelial cells (NESCs).

**N1437S**: Fibroblast cell studies in the N1437H variant PD patients do not show any change in cell adhesion patterns as compared to WT controls, including upon kinase inhibition [57].

**R1441C**: Sanders and colleagues. reported a significant increase in mitochondrial DNA (mtDNA) damage in iPSC-derived NPCs (Neural Progenitor Cells) and neural cells from patients heterozygous for the LRRK2 R1441C mutation compared to WT controls [58], a phenotype that can be rescued by Zinc Finger nuclease (ZFN)-mediated genome editing [58].

The R1441C mutant displays a reduction in GCase activity via a RAB10 regulated pathway in patient-derived fibroblast and iPSC-derived DA neurons, which is restored on treatment with the LRRK2 kinase inhibitor MLi2 or the over-expression of Rab10. The authors also reported a reduction in GCase activity in the iPSC-derived neural cells in comparison to WT controls [59]. Furthermore, the evidence from pre-symptomatic PD patient-derived reprogrammed neural cell populations indicate that the R1441C mutation results in the cells showing differential vulnerability to cellular stressors (valinomycin, concanamycin, and MPP^+^). They also show a reduced oxygen consumption, increased susceptibility to oxidative stress, and a dysfunctional mitochondrial mobility as compared to healthy controls [60].

**R1441G**: A study by Bahnassawy and colleagues. suggests that LRRK2 is integral to the regulation of cellular oxidative stress management, which is necessary for the survival of Neural Stem Cells (NSCs), while the pathogenic R1441G mutant results in a down regulation of the same pathway [61].

In 2016, it was reported for the first time that the LRRK2 R1441G (prevalent in the Basque populations) derived iPSCs were studied to compare α-synuclein propagation in the mature mutant neurons in which no change was observed in α-synuclein levels as compared to WT neurons [62]. Nevertheless, knockdown of LRRK2 caused a significant reduction of α-synuclein levels overall and a marked decrease was also reported in the R1441G mutant. Finally, the authors also report a differential regulation of the NF-ĸB transcriptional targets.

#### 2.2.3. ROC—Cellular and Molecular Effects of the Mutation in Experimental Models

**R1398H**: Molecular modelling provides indications of an increase in the intermolecular ROC-COR dimerization due to the R1398H variant. In vitro studies also suggest that while the GTP binding is not completely abolished, the R1398H variant results in about a 47% decrease in GTP binding as compared to the WT LRRK2. The study also indicates a stark increase in steady state GTP hydrolysis and an increase in the canonical Wnt signaling. Moreover, an increased axon length is observed in rat primary cortical neuron cultures expressing the R1398H mutant compared to WT [63].

**N1437H**: Analysis of this mutant shows that it can be locked in the dimeric conformation and it displays reduced GTPase activity [64]. While this mutant displays a kinase activity comparable to LRRK2 WT, it differs from LRRK2 WT in that its GTP-binding is enhanced, phosphorylation at S935 is reduced, and its subcellular distribution in the cytoplasm is less diffuse than LRRK2-WT and displays filamentous accumulations [65].

**R1441C**: The pathogenic R1441C mutant shows decreased GTPase activity compared to WT [21,24]. Interestingly, LRRK2-R1441C displays a reduced basal phosphorylation at the phosphosite cluster in the ANK-LRR interdomain region, but to a much weaker extent than LRRK2-R1441G [66,67].

In a study by Tong and colleagues exploring the effects of R1441C mutation in a mouse Knock-In (KI) model, the homozygous LRRK2 R1441C KI mice appeared mostly normal when subjected to spontaneous locomotor activity and motor coordination tests. The morphology of DA neurons and projections appeared normal, and no significant difference in the number of DA neurons or dopamine levels observed between KI mice and WT controls (3, 12, 23 months) [68]. Nevertheless, the mice displayed reduced amphetamine (AMPH)-induced locomotor activity and stimulated catecholamine release in cultured chromaffin cells. This mutation also impaired dopamine neurotransmission and D2 receptor function [68].

**R1441G**: LRRK2-R1441G leads to reduced GTPase activity, increased kinase activity, decreased dimerization [24], and reduced phosphorylation at the ANK-LRR interdomain region [66]. Increased tau hyperphosphorylation has been reported in the brain tissue of LRRK2-R1441G BAC transgenic mice when compared to the WT LRRK2 counterpart [69].

**R1441H**: The R1441H mutation displays an increased GTP binding affinity and reduction in GTP hydrolysis activity [70]. Overall, ROC domain pathogenic mutants show reduced GTPase activity, increased residence time of the GTP-bound “active state,” and increased kinase activity, compatible with a gain of function associated with PD pathogenesis.

### 2.3. COR Domain

The variants in the COR domain include the pathogenic mutation Y1699C as well as the risk factor mutations R1628P and S1647T.

#### 2.3.1. COR—Patient Characteristics

**R1628P** and **S1647T**: In a longitudinal follow-up study, S1628P and S1647T carriers showed a greater UPDRS motor score progression than patients not carrying LRRK2 mutations [71].

**M1646T**: In a study by Sosero and colleagues [72], statistical analysis was used to depict that the M1646T mutant increased the risk for PD with small effect and studies from peripheral blood cells also showed an increased GCase activity, which is unlike the R1441C/G and G2019S LRRK2 mutants.

**Y1699C**: A very detailed clinical and neuropathological description of the Y1699C missense variant carriers was provided by Khan and colleagues [73]. The Y1699C mutation was studied in the Lincolnshire kindred from England. The mean age of onset is 50 years; one of the living subjects reported having a unilateral tremor in one leg while being seated, whereas certain patients reported foot dystonia, other motor features, and few complaints of freezing episodes as well as orthostatic hypotension, and mild degrees of cognitive impairment. Nicholl DJ [74] reported nigrostriatal dysfunction via a PET scan, and an altered ^19^F-Dopa uptake was observed, typical of idiopathic PD (this was reported prior to the identification of the Y1699C mutation). Neuropathological examination of the PD brain of one subject shows no atrophy, reduced size of lateral ventricle as a result of brain swelling (consequence of hypoxia), and extreme pallor of the SNpc, while further histopathological examination depicted an acute neuronal loss in the SNpc, marked gliosis, LB pathology, α-syn and LN, mild Purkinje cell loss with empty baskets, and occasional axonal torpedos on neurofilament immunohistochemistry was also reported, The LC was positive for LB and LN, which was also sparsely present in the olfactory bulb. Neurofibrillary tangles and Aβ were also reported in various parts of the brain [74].

**S1761R**: Lorenzo-Betancor and colleagues published a study depicting a novel missense mutation in the highly conserved (prokaryotes and eukaryotes) Serine 1761 residue by screening late-onset PD (LOPD) patients with no known LRRK2 mutations. The S1761R mutant was observed in a large family with LOPD as well as in two independent EOPD patients, confirming the mutant being responsible for PD. This mutation is associated with typical PD, with patients exhibiting asymmetrical onset of motor features, mild resting tremor, but no definitive age of onset. Patients also responded well to Levodopa and displayed typical levodopa induced complications [75]. However, Mata and colleagues later published a letter contradicting the data stating that the S1761R is not to be considered a pathogenic mutant [76].

#### 2.3.2. COR—Patient-Derived Cell Characteristics

**R1628P and Y1699C**: Cells derived from patients carrying these two mutations have yet to be described at the time of writing this manuscript.

**S1647T**: iPSCs have been generated from S1647T mutations carriers. However, the characterization phenotype has yet to be established [77].

#### 2.3.3. COR—Cellular and Molecular Effects of the Mutation in Experimental Models

**Y1699C**: LRRK2-Y1699C displays a strong to moderate increase in kinase activity and an increase in GTP-binding activity, implicating the COR domain as a potential mediator of GTPase and kinase activity [78]. In a detailed report on the molecular mechanisms of this mutant in cellular models published by Daniëls and colleagues [24], a reduced LRRK2 GTPase activity, weakened ROC-COR dimerization, and strengthened intra-molecular ROC:COR interaction was reported. As has been described for several of the LRRK2 pathogenic mutants, overexpression of the Y1699C mutant of LRRK2 in primary neurons leads to a reduction in the neurite outgrowth [79].

### 2.4. Kinase Domain

D1887G, G2019S, I2020T

#### 2.4.1. Kinase—Patient Characteristics

**D1887G**: A study of two PD patients carrying a D1887G mutation, patient A (Female [**F**]) and patient B (Male [**M**]) with an age of onset of forty-eight and fifty was done. The patients presented with rigidity and bradykinesia and non-motor symptoms, which included insomnia, dysarthria, and blepharospasm [patient A] and erectile dysfunction, mania, hyper sexuality, and insomnia [patient B] [46].

**G2019S**: The G2019S mutation is among the most widespread of the LRRK2 mutations. Indeed, a meta-analyses of 68 studies from 32 countries showed that the G2019S mutation is present throughout the globe with frequencies of 1% overall of PD cases, 3–6% in familial PD in Europe, and up to 42% in specific populations such as North African Arabs [80]. The cumulative risk of PD by the age of 59 years ranges from 13–45% [81,82], from 21–85% at 69 years [82], with the widest range of 32% to 100% at 79 years [83]. The main clinical features of patients with LRRK2 G2019S-associated PD include asymmetrical, tremor-predominant Parkinsonism, bradykinesia, rigidity that is mitigated by dopamine replacement or sanative neurosurgery, and non-motor symptoms, which makes it difficult to clearly distinguish between familial PD affected individuals and idiopathic cases. However, the G2019S carriers displayed an abduction–adduction leg tremor that was markedly more prevalent than in the idiopathic patients [84]. In comparison to idiopathic PD patients, G2019S mutation carriers also display a reduced risk of cognitive impairment, hyposmia, a longer time for their first fall, and a higher propensity for dystonia. Patients also experienced a less aggressive manifestation of the symptoms, late requirement of dopamine-replacement therapies, but were more prone to drug-related dyskinesia [73,85]. Neuropathological and histopathological studies show a typical loss of neurons in the SNpc and *LC*, LB pathology, increased tau phosphorylation, presence of neurofibrillary tangles [86,87]. Interestingly, the G2019S mutation is more frequently associated with LB pathology compared to other LRRK2 mutations [88]. In terms of the severity of disease, there was no gene dosage effect observed, and G2019S carriers showed a more benign disease course [89]. In terms of biological readouts, some limited information is available from the analysis of biosamples from G2019S carriers. For instance, PBMCs from LRRK2 G2019S PD patients show a reduction of the S935 phosphorylation compared to PBMCs from idiopathic patients [90]. Additionally, studies in urinary exosomes have shown that total LRRK2 and phospho-Ser1292-LRRK2 are increased in LRRK2 G2019S carriers [91]. In addition, a recent proteomics study of urine samples from LRRK2 G2019S carriers identified several urinary proteins that discriminate LRRK2 G2019S carriers from controls, as well as potential indicators for disease manifestation in these carriers, including VGF [92].

**I2020T**: The first clinical characteristics provided by Nukada and colleagues in 1978 described the Sagamihara kindred that would later be found as carrying the LRRK2 I2020T variant [93,94]. Hasegawa reported that the I2020T variant disease prognosis is almost indistinguishable from that of the sporadic PD patient in terms of age of onset (38–74 years), with patients presenting with resting tremor (less frequent as compared to other cohorts of PD patients) and gait disturbances (more frequent than sporadic PD patients and other PARK8 variant carriers). The patients were also responsive to levodopa therapy, but showed motor fluctuations associated with long-term use of Levodopa. They complained of altered olfactory function and ^123^I-MIBG uptake. Cognitive deficiencies and depression were rather rare with mean disease duration lasting about 25 years, with average lifespans a little greater than 70 [95]. Ujiie and colleagues described a confirmed I2020T variant levodopa responsive PD patient, a 68-year-old female, who had a disease progression of 17 years, ultimately succumbing to multiple organ failure due to pneumonia-related complications. The patient presented with clumsiness in the legs at the age of 51 with pronounced gait disturbances; at 57, she suffered a “wearing off” motor fluctuation; visual hallucinations by age 64, and by the age of 65, required assistance to walk [96].

#### 2.4.2. Kinase—Patient Cell Derived Characteristics

A robust two-fold elevation of the α-synuclein levels was reported in iPSC-derived DA neurons with the LRRK2 G2019S mutation [62]. The G2019S mutant neurons depict a dysregulation of genes that have been implicated in neurodegeneration and also increase extracellular signal regulated kinase 1/2 (ERK1/2) phosphoregulation; the mutant neurons also have an altered Ca^++^ homeostasis, which renders the neurons vulnerable to ER stressors [97,98]. In 2011, Nguyen and colleagues reported an elevated expression of stress response genes, altered α-synuclein levels, and increased vulnerability to neurotoxins, which is limited to TH^+^ cells (DA neurons) [99]. Recently, a study by Schwab and colleagues reported the G2019S DA neurons with impaired mitochondrial localization, trafficking patterns, and cell-specific bio-energetic modifications [100]. These results have also been described in detail by Singh and colleagues in a recent review [101]. Neuroepithelial cell (NESCs) studies by Walter and colleagues show an increase in fragmented mitochondria (generally a good mitochondrial fusion/fission ratio is maintained), robust increase in Reactive Oxygen Species (ROS), decreased basal respiration compared to WT NESCs, a reduction in membrane potential, and a significant reduction in mitophagic clearance by lysosomes. All these characteristics contribute to the cell death that is associated with disease [102]. On the contrary, Yakhine-Diop and colleagues reported an increased clearance of defective mitochondria in G2019S fibroblasts. The study also investigated the variation in autophagy enzymes Histone acetyltransferase (HAT) and histone deacetylase (HDAC) with G2019S idiopathic PD (IPD) fibroblasts and concluded that a reduction in mitophagy and HDAC activity is observed in IPD fibroblasts [103]. Another study reported amplified mitophagy and loss in mitochondrial membrane potential, reduced mitochondrial mass, and reduced citrate synthase activity with increased autophagic flux in G2019S patient fibroblast cells [104]. A study by Mortiboys and colleagues depicts a reduction in mitochondrial membrane potential in G2019S fibroblast cells, reduced ATP levels, and an increase in elongation and interconnectivity of mitochondria. The study also concludes that it is unclear whether this is an effect of kinase overactivity or a consequence of haploinsufficiency [105]. A study by Juárez-Flores reported an enhanced mitochondrial performance and autophagic flux in pre-symptomatic G2019S fibroblast in comparison to healthy controls and G2019S patients; prolonged upregulation of mitochondrial performance could potentially drive the accelerated death of DA neurons on the long run and contribute to disease [106].

In a more therapeutic-based approach to determine the relationship between astrocytes and G2019S patient-derived ventral midbrain DA neurons (vmDAns), di Domenico and colleagues set up an experiment to study the potential role that chaperone-mediated autophagy (CMA) could play in clearance of α-synuclein. Interestingly, they were able to report an increased clearance of α-synuclein from G2019S vmDAns co-cultured with control astrocytes on treatment with CMA enhancers. They also propose an alternate strategy that aims to block the cross-talk between the different neural populations to delay the spread of the pathogenic α-synuclein that contributes to disease [107,108].

Another therapeutic strategy proposed by Hockey and colleagues is based on their results, which indicate that G2019S patient-derived fibroblast cells have enlarged lysosomes, aggregated around the nucleus, and could be characterized as heterogenous translucent areas via microscopy, a phenotype that was reversed by inhibition of the LRRK2 kinase domain, silencing of TCP2, a ubiquitous protein implicated in disease, and pharmacological inhibition of the TCP regulators. The study also proposes that TCP2 could be a substrate for LRRK2 phosphorylation [109]. A study published by Caesar and colleagues in 2014 shows an increase in anti-inflammatory, neurotrophic protein progranulin (PGRN); this protein is said to be increased in the CSF collected from the LRRK2 G2019S PD patients and decreased in the supernatants of cultured G2019S fibroblast cells collected from pre-symptomatic individuals (unchanged mitochondrial function)[110]. This study was followed by another paper from the same group suggesting that LRRK2 G2019S alters actin dynamics and F-actin in patient-derived fibroblasts [111].

Studies in EBV transformed Lymphoblastoid cells (LCLs) by *Dzamko* and colleagues in 2010 showed that G2019S derived LCLs depicted a three-fold increase in kinase activity, with equal levels of phosphorylation of the S910 and S935 sites between WT and G2019S variants; however, potent inhibition of the G2019S variant was observed on these sites post treatment with a kinase inhibitors, along with characteristic loss in 14-3-3 binding and relocalization of LRRK2 in the cytoplasm [112]. A study in 2017 by Howlett and colleagues showed an increase in mtDNA damage in G2019S LCLs as compared to age-matched controls, but not in G2019S fibroblast cells [113]. Recently, centrosomal cohesion deficits in fibroblasts as well as in LCLs derived from G2019S PD patients, which could potentially serve as a biomarker in PD diagnosis, have been reported [114].

Beyond their use as homogeneous cultures, iPSCs also allow the generation of organoid cultures, literally adding another dimension to the investigation of disease progression and pathogenesis in patient cell culture. Smits and colleagues used midbrain floor plate neural progenitor cells to create organoids that demonstrated dopamine-producing and -secreting midbrain neurons. The authors of this study observed that the organoids generated from individuals harboring the LRRK2-G2019S mutation showed reduced number and complexity of the midbrain DA neurons compared to controls [115]. These PD-relevant phenotypes suggest that the implementation of organoid cultures is relevant to study PD pathomechanisms. In the event that a correlation could be made between findings in patient cells and findings in patients, then patient cells may be useful as a tool to test whether specific pharmacological therapies show good responses in specific individuals. This approach has already been used to assess activity of LRRK2 kinase inhibitors in patient-derived blood cells [116,117] and suggests that personalized medicine approaches are also feasible in LRRK2 PD.

#### 2.4.3. Kinase—Cellular and Molecular Effects of the Mutation in Experimental Models

**G2019S**: The first indications of how the G2019S mutation is affecting LRRK2 function came from the analysis of the protein sequence, where it was found that G2019S constitutes a modification of the DYG activation loop (the DYG motif is a small conserved sequence in the LRRK2 sequence that enables it to flexibly switch between the active and inactive state of kinase activity [118]) in the LRRK2 kinase domain (reviewed in [119]). Enzymatic testing of the LRRK2 G2019S mutant protein later revealed that this modification leads to enhanced kinase activity [120,121]. Overexpression of this mutant in cell culture as well as in vivo showed adverse effects on viability that were reversed when the G2019S mutant is combined with a kinase inactivating mutation [120,122,123]. In 2006, MacLeod and colleagues demonstrated that the G2019S mutant significantly reduced neurite length and branching as well as had tau-positive inclusions in primary neurons and the rodent CNS system. However, the LRRK2-deficient systems displayed an increase in neurite length and branching [124].

In 2009, Parisiadou and colleagues studied the relationship between ERM (Ezrin, radixin, and Moesin) proteins and LRRK2 on neuromorphology. ERM proteins have previously been described as physiological substrates of LRRK2 [125] and play a pivotal role in maintaining cell shape, growth, and motility by interacting with filamentous actin. They concluded in this study that the inhibition of the ERM proteins in the G2019S mutants could potentially reverse the neuronal defects observed by MacLeod and colleagues [41,124].

A study by Volpicelli-Daley and colleagues [126] reports an increase in the recruitment of endogenous α-synuclein into inclusions on exposure to α-synuclein fibrils in both cultured primary mouse neurons and DA neurons cultured from rat SNpc. Further WT-LRRK2 overexpression was shown to reduce the abundance of the inclusions. The authors also blocked the effects of the G2019S LRRK2 mutant on inclusion formation by treating the primary neurons with various kinase inhibitors. Further evidence of LRRK2 dependent alteration in α-synuclein levels influencing inclusion formation was provided when a knockdown of total α-synuclein by antisense oligonucleotides reduced the inclusion formation in G2019S neurons. These reports suggest that the G2019S LRRK2 has the potential to increase the pathological inclusion formation by exacerbating the pooling of α-synuclein, which is more prone to forming the problematic inclusion bodies. This premise is consistent with the results from Hu and colleagues showing that the G2019S LRRK2 mutant promoted the aggregation of α-synuclein, inhibited the degradation of α-synuclein in stably transfected HEK293T cells, and reduced lysosomal enzymatic activity and decreased activity of cathepsin B and cathepsin L [127].

Over the years, studies have aimed to determine the role of LRRK2 in the healthy functioning of the motor circuit by determining behavioral alterations, alterations to the dopaminergic system, and carrying out neuropathological examination. For instance, in a 2012 study by Hinkle and colleagues [128], LRRK2 Knockout (KO) mice that lacked exon 41 (responsible for encoding the kinase domain activation hinge of LRRK2) were extensively studied for up to 20 months of age for their dopamine dynamics, behavioral patterns, and neurogenesis analysis. Even though their study reported that there was no significant changes observed in the striatal system and the dopamine transport dynamics, the LRRK2 KO mice showed significantly different behavioral patterns [128]. In another study from the same group [129], G2019S KI mice were reported to have elevated kinase activity in the brain of both homozygous and heterozygous mutation carriers, impaired dopamine dynamics by 12 months of age in both cohorts, and significant mitochondrial abnormalities in the striatum of older homozygous G2019S mice. A study by Xiong and colleagues in transgenic mice [130] reports that the overexpression of LRRK2 G2019S in mouse forebrain results in behavioral deficits and an α-synuclein pathology characteristic of LRRK2 G2019S PD. Longo and colleagues reported that treatment of G2019S KI mice with kinase inhibitors reversed the hyperkinetic phenotype observed in these models [131].

**I2020T**: The 12020T mutant lies in the kinase domain of the LRRK2 protein. The effects of I2020T have been less clear than for the G2019S mutant, with both increases and decreases in kinase activity reported in different studies. However the initial report on the I2020T mutation from Gloeckner and colleagues [132] suggested a significant increase in autophosphorylation (by 40%) in comparison to the wild-type LRRK2 in vitro. The I2020T mutant also does not hamper the GTPase activity of LRRK2 [132]. In 2015, It was reported that the neurons from reprogrammed iPSCs from the original Sagamihara kindred [87] with I2020T mutation displayed reduced phospho-AKT levels, increased apoptosis, increased glycogen synthase kinase 3-β, and increased tau phosphorylation. In line with what is observed with other mutants, the expression of LRRK2 I2020T leads to a reduction in neurite length in primary neurons [124].

### 2.5. WD-40 Domain

#### 2.5.1. WD40—Patient Characteristics

**G2294R**: The LRRK2 G2294R was reported in a 67-year-old patient of Japanese origin, with akinesia, resting tremor, and decent response to levodopa carbidopa. The patient met the standard clinical criteria for PD, and again at 70 years of age, the patient reported auditory hallucinations while sleeping, gait disturbances, and pain in the toes during the off-period. The patient had a Movement Disorder Society-Unified Parkinson’s Disease Rating Scale (MDS-UPDRS) score of 13 (Part I, 6;Part II, 1; Part III, 2; Part IV, 4) at the on-period, Hoehn and Yahr stage indicated 0 at the on-period and 1 at the off-period, cognitive function score of 30 on 30 on the Mini-Mental State Examination (MMSE), a 30 of 30 on the revised Hasegawa’s dementia scale, and 13 on 18 on the Frontal Assessment Battery [133].

**G2385R**: The LRRK2 G2385R was first identified as a functionally relevant variant, acting as a common risk factor for sporadic PD in the Chinese population [134]. Another interesting study published by Tan and colleagues nominated the G2385R variant as a risk factor for sporadic PD in Asian populations after conducting a detailed pooled analysis of individual studies [134,135,136]. The age of onset for the G2385R variant carriers lies between the ages of 39 and 79 [137]. G2385R carrier patients have clinical signs similar to idiopathic PD patients, with some differences such as a higher MMSE scores, lower Hoehn and Yahr rating, higher levodopa equivalent daily dose (LEDD), and higher likelihood of developing motor symptoms compared to non-carriers [138].

#### 2.5.2. WD40—Patient-Derived Cell Characteristics

**G2294R**: LRRK2 protein levels are reduced in macrophages that were differentiated from monocytes derived from the LRRK2 G2294R variant patient [133].

#### 2.5.3. WD40—Cellular and Molecular Effects of the Mutation in Experimental Models

**G2385R**: It has been well documented that the WD40 domain plays a role in the protein-protein interaction function of LRRK2, evidence from studies also suggest that the WD40 domain is the key to LRRK2-mediated cell death, which could result in PD. The G2385R variant has been long associated with reduced kinase activity of LRRK2 and auto-phosphorylation in vitro [139]. However, it has only recently been characterized as a loss of function mutation, resulting in an impaired interaction between LRRK2 and synaptic vesicles, due to reduced binding efficiency with actin and synaptin (presynaptic proteins) [140]. The G2385R mutation also results in a higher affinity of LRRK2 for proteins involved in proteasomal degradation Hsc70 and carboxyl-terminus of Hsc70-interacting protein (CHIP), thereby favoring LRRK2 degradation [141].

**Table 1:** An overview of phenotypes observed in cells cultured from patients carrying LRRK2 mutations. Phenotypes of patient-derived cells from individuals carrying LRRK2 mutations as described in published studies are summarized here. Each line of the table corresponds to a single mutation in a single cell type from a single study and provides the test characteristics and main phenotypes observed.

	Cell Types	Nature of Patient-Derived Cells	Patient Cell Phenotypes	Reference
**N1437S**				
1.	Fibroblast cells	Skin biopsies obtained from four healthy, two N1437S PD patients, three G2019S patients and one R1441C patient.	No change was observed in cell adhesion between patient fibroblast and control cells in both basal and kinase inhibited state.	[57]
**R1441C**				
2.	DA neurons	Fibroblast cells (from the Northwestern University Biorepository and NINDS human cell biorepository) were reprogrammed to iPSCs and differentiated to DA neurons.	The LRRK2 R1441C mutant depicted a reduction in lysosome specific GCase activity. The knockdown of Rab10 (*bona fide* substrate of LRRK2) resulted in reduced GCase activity. The R1441C mutant results in an increased level of phospho-Rab10 and over-expression of Rab10 increases the GCase activity.	[59]
3.	Neural cells derived from iPSCs	The iPSC lines are derived from 2 (Twins—brother and sister) heterozygous LRRK2 R1441C (gene from father with PD) carriers. No clinical manifestation of PD in sister, however resting tremor observed in brother.	Lactate dehydrogenase (LDH) enzyme release was used in order to measure neuronal cell vulnerability, MTS assay— LDH/MTS assays, immunocytochemistry and cell counts depicted an increased vulnerability of iPSC derived neural cells from R1441C variant carriers on exposure to chemical stressors (concanamycin A, valinomycin), reduced oxygen consumption and dysfunctional mitochondrial mobility as compared to healthy controls.	[60]
4.		The iPSCs from at risk PD related R1441C variant carriers were obtained from the Coriell BioBank.	Significant mtDNA damage observed in iPSC cell lines differentiated into neural cells using two separate protocols.	[58]
5.	Fibroblast cells	Skin biopsies obtained from four healthy subjects as well as, two N1437S, three G2019S and one R1441C PD patients.	No change was observed in cell adhesion between patient fibroblast and control cells in both basal and kinase inhibited state.	[57]
**R1441G**				
6.	DA neurons	Fibroblast cells (from the Northwestern University Biorepository and NINDS human cell biorepository) were reprogrammed to iPSCs and differentiated to DA neurons.	A reduction in lysosome specific GCase activity was reported.	[59]
7.		Fibroblast from PD patients with R1441G mutation in LRRK2 (*n* = 2) and matched healthy subjects. Controls include Human embryonic stem cell line (H9), so as to study effects of the lentiviral reprogramming on the cells.	This study was the first to report an iPSC line with the R1441G mutation. α-synuclein levels remained unchanged in the R1441G differentiated mature DA neurons as compared to controls.	[62]
**G2019S**				
8.	Neuro-progenitor cells and mature neural cells derived from iPSCs	Patient-derived fibroblasts are reprogrammed into iPSCs, three homozygous/heterozygous LRRK2 G2019S patient, three age matched healthy subjects w/o LRRK2 mutations	Significant mtDNA (mitochondrial DNA) damage observed in iPSC derived NPC and neural cells with G2019S mutation and not in the fibroblast cells or undifferentiated iPSCs. Indicating that mtDNA damage might be a neural specific phenotype.	[58]
9.	Neural cells derived from iPSCs	The iPSC lines from 2 heterozygous R1441C variant carrier, 1 homozygous PD patient and healthy controls were used in this study	Lactate dehydrogenase (LDH) enzyme release was used in order to measure neuronal cell vulnerability, The G2019S mutant cells showed increased vulnerability to valinomycin, concanamycin and MPP^+^ (selected stressors directed to assess mitochondrial function or protein degradation) in neural cells in comparison to control fibroblast cells.	[60]
10.	Neurons differentiated from iPSCs	Fibroblast from two patients harboring the LRRK2 G2019S mutations reprogrammed into iPSCs, further, differentiated into neurons. Nonisogenic healthy controls iPSCs were generated from healthy women.	The authors reported a prominent dysregulation by means of alterations in gene expression of *CPNE8* (role in calcium mediated intracellular processes), *MAP7* (involved in microtubule dynamics), *UHRF2* (involved in cell-cycle regulation), *ANXA1* (vital role in phospholipid binding), ***CADPS2*** (involved in exocytosis of vesicles with neurotransmitters/neuropeptides), in differentiated neurons out of which 4 genes are known contributors of DA neurodegeneration. The mutation also results in an increase in extracellular signal regulated kinase 1/2 (ERK) phosphorylation	[97]
11.		PD patient LRRK2 heterozygous G2019S iPSCs derived neurons and tested against control.	The study concludes that the LRRK2 plays a pivotal role in the Endoplasmic reticulum (ER) Ca^2+^ homeostasis, the upregulated kinase activity of the G2019S mutant. Alteration of calcium homeostasis along with ER stressors potentially result in the neurite collapse.	[142]
12.	Midbrain DA neurons derived from iPSCs	Fibroblast cells (from the Northwestern University Biorepository and NINDS human cell biorepository) were reprogrammed to iPSCs and differentiated to DA neurons.	Significant a reduction in the GCase activity of LRRK2 G2019S iPSC neuronal cells in comparison to the healthy controls, which was reversed on correction of the mutation using CRISPR-Cas9.	[59]
13.		Fibroblast from 2 PD patients with G2019S mutation in LRRK2 and matched healthy subjects. Controls include Human embryonic stem cell like cells (H9)	α-synuclein levels were two-fold higher in the G2019S differentiated mature DA neurons than controls. Marked increase in autophagic mediator p62 (responsible for clearance of α-synuclein). The G2019S mutant results in an impaired NF-κB signaling and the gene transcription of NF-κB was altered in LRRK2 knockdown experiments.	[62]
14.		G2019S iPSCs generated from dermal fibroblast cells. Further differentiated into midbrain DA neurons.	No difference was observed between differentiated and undifferentiated iPSC cell from the G2019S and WT iPSCs and hESCs in terms of pluripotency markers, general morphology, differentiation potential, epigenetics and gene expression profiles. G2019S affected neurons displayed higher than normal expression of stress response genes altered α-synuclein proteins accumulations and increased vulnerability of neurons to neurotoxins (compared to unaffected controls).	[99]
15.	iPSCs- derived DA, glutaminergic and sensory neurons	Human iPSCs were obtained from two homozygous LRRK2 G2019S patient cells, one heterozygous G2019S carrier and three healthy controls from publicly available samples from NINDS Coriell Institute.	The study indicates that the G2019S results in an impaired mitochondrial respiration pattern in the iPSC derived DA and glutaminergic neurons, however, sensory neurons showed no such trends. The G2019S iPSC derived DA neurons displayed alternate mitochondrial distribution and trafficking patterns as well as cell specific bio-energetic modifications caused by reduced NAD^+^ and sirturin deacetylatse which is quite indicative of a disease specific phenotype.	[100]
16.	Neuro-epithelial cells (NESCs)	The study looked at thirteen human iPSC-derived NESC lines obtained from three patients carrying the LRRK2-G2019S mutation with four healthy individuals (age/gender matched). Four isogenic NESC lines were generated, two of which had a mutation introduced and the other two in which the mutation was corrected.	Patient NESC have an increased number of fragmented mitochondria, reduced membrane potential and reduced mitophagic clearance via lysosomes in comparison to isogenic control lines.	[102]
17.	Astrocytes	A Co-culture of iPSC derived astrocytes and ventral midbrain DA neurons (vmDAns) from familial G2019S PD patients	Chemical enhancement of Chaperone Mediated autophagy (CMA) protected PD astrocytes and vmDAns via clearance of α-synuclein accumulations. Non-cell autonomous contribution of astrocytes during PD pathogenesis. Possibility of exploring a therapeutic strategy of blocking pathogenic cross talk between neurons and glial cells. Dysfunctional CMA (chaperone mediated autophagy), impaired macroautophagy, progressive α-synuclein accumulation.	[107]
18.	Fibroblast cells	3 skin fibroblast groups - 1) Control (Patients who did not develop PD) 2) Idiopathic PD—IPD (Patients without G2019S PD) 3) G2019S PD patients The overall goal of the study is to examine variation in the levels of histone acetyltransferase (HAT) and histone deacetylase (HDAC), described as the key players of autophagy.	The fibroblasts from G2019S cells display an increased clearance of defective mitochondria where as those of IPD show a reduction of mitophagy and increased ROS. The acetylation proteins between the two groups vary, however, HDAC activity is reduced in IPD cells (HAT activity unchanged), the imbalance of autophagy enzymes leads to cell death. The inhibition of the HATs in IPD cell lines could be considered as cyto-protective in this case.	[103]
19.		Skin biopsies collected from 5 individuals of confirmed G2019S PD diagnosis, results compared to 5 healthy age matched controls.	1. Mitochondrial membrane potential (decrease by 45%) and ATP levels are reduced in LRRK2 mutant fibroblast cells 2. Increased elongation and interconnectivity of mitochondria in LRRK2 mutant patient cells.	[105]
20.		Skin biopsies donated by four G2019S PD patients and four healthy controls.	Lysosomes were enlarged/swollen, through microscopy they could be characterized by large translucent areas and clustered around the nucleus in G2019S patient-derived fibroblasts. These defects were reversed by inhibition of kinase activity using LRRK2In1, silencing of TPC2 (Two pore channels—acidic vesicles that comprise the endolysosomal system) and pharmacological inhibition of TPC regulators [Rab7, NAADP and PtdIns (3,5)*P*_2_] crucial to autophagy.	[109]
21.		Fibroblasts cultured from punch skin biopsies obtained from nine presymptomatic PD G2019S mutation carriers and age matched healthy controls	A reduction in the secretion of Progranulin (PGRN) was observed in supernatants of cultured human fibroblasts isolated from presymptomatic LRRK2 (G2019S) mutation carriers, and mitochondrial function remained unaffected.	[110]
22.		Fibroblast cells were obtained via skin biopsies from LRRK2 G2019S mutation carriers both with and without PD as well as healthy controls.	Cells from the G2019S mutant carriers w/o symptoms show an enhanced mitochondrial performance as well as upregulated autophagic flux in comparison to healthy controls and G2019S patients. Therefore, the hyperactivity of mitochondrial complex as well as autophagy could be the driving force for carriers to develop symptoms.	[106]
23.		Two separate (1 male, 1 female) LRRK2 G2019S PD patient fibroblast cell lines were obtained from the Coriell biorepository	Amplified mitophagy is observed in patient fibroblasts as compared to healthy controls, this is further supported by the evidence indicating a significant loss of mitochondrial membrane potential, reduction in mitochondrial mass and reduced citrate synthase activity accompanied by increased autophagic flux. These results indicate that G2019S mutant accelerates autophagy in cells.	[104]
24.		Skin biopsies were obtained from four healthy, two N1437S PD patients, three G2019S patients and one R1441C patient.	The G2019S mutant did not result in any change in cell adhesion patterns in fibroblast cells as compared to healthy control fibroblasts, neither in basal conditions nor in conditions of inhibition of LRRK2 kinase activity.	[57]
25.		Skin biopsies from three PD G2019S patients and four healthy individuals were obtained	An increase in kinase activity and autophagic flux in the mutant fibroblast cells was observed. However, MEK/ERK (MAPK signaling pathway) inhibition reduced the autophagic flux and sensitivity of the G2019S mutants in comparison to healthy controls.	[143]
26.		Skin biopsies obtained from G2019S mutation carriers (not directly related to each other) without clinical symptoms of PD. Control fibroblasts obtained from age/gender matched controls (*n* = 5)	Increased sensitivity of LRRK2 mutant fibroblasts to LatA (drug known to disrupt microfilament organization, cell shape [144]). They also observe a significant increase in F-Actin bundles with a decrease in filopodial length. This depicts a pattern of LRRK2 mutant dependent alteration of F-Actin dynamics.	[111]
27.	Lymphoblastoid cells (LCLs)	LCLs samples from six patients with heterozygous G2019S mutation, thirteen sporadic PD patients and thirteen unrelated gender/age matched controls. For the study additionally three gender/age matched LCL controls, six gender/age matched LCLs from heterozygous G2019S LRRK2 patients were obtained from the NINDS Coriell Cell Repository.	The authors concluded that the G2019S LCLs and a subset of LCLs derived from sporadic PD patients displayed a centrosomal cohesion deficit, reverted by LRRK2 kinase inhibition.	[114]
28.		Endogenous LRRK2 activity was studied in EBV-transformed LCLs derived from a PD patient with homozygous G2019S mutation and one healthy control (no LRRK2 mutations)	LRRK2 levels were similar in both cell lines, however, a three-fold increase in kinase activity was observed in the G2019S mutant. The S910, S935 sites in both the mutant and healthy LCLs were equally phosphorylated. However, on treatment with kinase inhibitors (H-1152, Sunitib), the phosphorylation of S910, S935 was more potently inhibited in the G2019S mutant than the healthy control.	[112]
29.		Six iPSC line derived LCLs and healthy age matched controls were used for the study (Parkinson institute or NINDS Coriell biorepository). Further, skin biopsies from three G2019S PD patient and three healthy individuals were obtained to study fibroblast cells.	An increase in the mtDNA damage was observed in LCLs of G2019S mutation carriers (with PD) as compared to LCLs from age matched healthy controls. However, no change was observed in mtDNA damage in fibroblast cells obtained from G2019S mutation carriers in comparison to healthy subjects.	[113]
**G2019S Organoids**				
30.	Midbrain floor plate neural progenitor cells (mfNPCs)	mfNPC are differentiated into 2D midbrain DA neurons (mDANs) and 3D human midbrain-specific organoids (hMOs) from PD patients carrying the LRRK2-G2019S mutation.	The expression of DA neuron markers *TH*, *AADC*, *VMAT2*, and *DAT* are markedly decreased in LRRK2-G2019S organoids as compared to the controls at day 60. Mature neuronal genes such as *NURR1*, *PITX3*, *EN1*, *TH*, and *MAPT* are also reduced in the LRRK2-G2019S mutant organoids but not in 2D cultures (where their expression is very low regardless of genotype). The DA neurons in LRRK2-G2019S organoids exhibited a decrease in neurite length.	[115]
**I2020T**				
31.	Neurons	I2020T PD patient fibroblast derived iPSCs were reprogrammed to neurons (Japanese Sagamihara kindred)	The I2020T neurons displayed reduced levels of phospho-AKT in comparison to control neurons, with an observable increase in apoptosis. An increase in glycogen synthase kinase-3β (GSK-3β)—a central intracellular regulator of vital functions—proliferation, migration, glucose metabolism) which is implicated in a number of diseases and high tau phosphorylation. Post-mortem histopathological studies reveal deposits of neurofibrillary tangles, increased tau phosphorylation of neurons.	[145]
**G2294R**				
32.	Peripheral blood mononuclear cells (PBMC)	Monocytes were isolated from PBMCs of G2294R PD patient and gender matched controls. Further monocytes were differentiated to macrophages	LRRK2 protein levels are increased in LRRK2 G2294R monocytes and reduced in macrophages. Further Rab8 and Rab10 levels were also decreased in macrophages.	[133]
**G2385R**				
33.	PBMC	PBMCs from G2385R patients were reprogrammed into iPSC cells and used to generate stable cell lines.	No characterization was reported.	[146]

Legend—An overview of phenotypes observed in cells cultured from patients carrying LRRK2 mutations. The studies have been grouped in order of the occurrence of the variant in the LRRK2 protein and the cell type. About 31 studies have been looked at to summarize patient-derived cell phenotypes. **iPSCs**—induced pluripotent stem cells, **DA**—Dopaminergic neurons, **PD**—Parkinson’s disease, **GCase**—glucocerebrosidase, **LDH**—Lactate dehydrogenase, MPP^+^, **WT**—Wild Type, **w/o**—Without, **mtDNA**—mitochondrial DNA, **NPC**—Neural Progenitor Cells, **ERK**—Extracellular regulated kinase 1/2, **ATP**—Adenosine Triphosphate, **BAC mice**—Bacterial Artificial Chromosome generated transgenic mice, **CMA**—Chaperone mediated autophagy, **EOPD**—Early onset Parkinson’s disease, **ER**—Endoplasmic Reticulum, **GCase**—Glucocerebrosidase, **GSK3β**—Glycogen synthase kinase 3 β, **H9 cells**—Human embryonic stem cell line, **HAT**—Histone acetylase, **HDAC**—Histone deactetylase, **hESCs**—Human Embryonic Stem Cells, **LCLs**—Lymphoblastoid cells, **LOPD**—Late onset Parkinson’s disease, **LRRK2**—Leucine rich repeat kinase 2, **MPP^+^**—1-methyl-4-phenylpyridinium (neurotoxin), **NAD^+^**—Nicotinamide adenine dinucleotide, **NESCs**—Neuro-epithelial cells, **NF-κB**—nuclear factor kappa-light-chain-enhancer of activated B cells, **PBMCs**—Peripheral Blood Mononuclear Cells.

## 3. Conclusions and Perspectives

The age of onset of PD and duration of disease vary significantly between each LRRK2 mutation, the patients generally present with classical manifestations of PD prior to further genetic screen that indicates variations in PD associated genes or in LRRK2 specifically. It should be noted that while some reports pinpoint subtle differences between sporadic PD patients and LRRK2 PD patients in specific test groups, the LRRK2 specific differences still fall within the general range of symptoms/phenotypes observed in idiopathic PD on a whole. Neuropathological studies of post-mortem brain samples from carriers of LRRK2 variants depict Lewy bodies, Lewy neurites, and depigmentation of the SNpc. Tau inclusions are also observed in a fraction of patients. Most LRRK2 patients are treated with dopamine replacement therapy, the response and tolerance of which widely varies among different patients. A number of similarities are observed in the clinical phenotypes presented by the LRRK2 mutation carriers when compared to idiopathic PD patients in the clinical setting, which makes it difficult to distinguish what observations constitute as differences between the two groups.

LRRK2 variants that have been included in the overview above are categorized under definitely pathogenic, possibly pathogenic, or rare variants. However, for some of the variants described, patient information is limited to a small number of patients, insufficient to conclusively represent the consequences of a variant. Therefore, larger cohorts of patients (and patient cells) with the same variants need to be identified and evaluated in order to draw solid conclusions on the phenotypes exhibited as a consequence of the variant. It should be noted that some additional insight may come from the study of LRRK2 variants that are not linked to PD but nonetheless affect LRRK2 function. For instance, in a case control study published by Blauwendraat and colleagues. Ref. [147], a large cohort of patients with LRRK2 loss of function variants, no significant increase in the risk of PD was identified, whereas a recent study by *Whiffin* and colleagues [148] reported reduced levels of LRRK2 protein in heterozygous loss of function protein coding genes, which are independent of specific phenotypes or state of disease.

In parallel to identifying clinical phenotypes, there is also a need to study patient-derived cell models to better understand the pathophysiological mechanisms of the different LRRK2 mutations in different cell types (not only neural cells, but also non-neural cells such as skin, kidney, lung, etc.). From the mutant per mutant trends described above and in Table 1, the important overall conclusion that can be made is that patient-derived cells harboring LRRK2 mutations display differences compared to control cell populations. These differences are variable from mutant to mutant but include differences such as an increase in LRRK2 kinase activity for the G2019S mutant, an increase in mitochondrial DNA damage, enhanced sensitivity to chemical stressors, and impaired lysosomal activity (see Table 1 for a full overview). One aspect of the interpretation of findings across different patient-derived cell types is to take into account the cell type under study. For instance, PD patient-derived fibroblasts with the G2019S and R1441C mutations did not show any change in cell adhesion patterns, actin cytoskeleton arrangement, or dynamics [57], despite the fact that these mutations show impaired neurite outgrowth in neuron cultures. This could potentially be linked to a neural cell-specific phenotype, which does not necessarily have profound effects on the skin cells. This example points to a balance that needs to be struck in pursuing studies in different types of patient-derived cells. On the one hand, shorter and less complex protocols for culturing patient-derived cells may provide a quick and economic way to obtain actionable data to inform patient treatment. On the other hand, more sophisticated and longer protocols allow for more flexibility and perhaps more possibilities for in-depth mechanistic studies. In that regard, it can be noted that besides differentiation protocols leading to homogeneous cultures, several other levels of sophistication can be added. Some examples include culturing in 3D (via the use of 3D matrices), neuron/glia co-cultures (for instance by combining iPSCs that are differentiated on the one hand to neurons and on the other hand to glia) [149], the reconstitution of key neuronal circuits such as the nigrostriatal pathway using microfluidics culture chips [150], or the use of organoid cultures. For example, work from Kim and colleagues described organoids generated from iPSCs with knock-in G2019S heterozygous mutation. The study reported an increased aggregation of α-synuclein, increased phosphorylation of α-synuclein at S129 and decreased DA neuron markers. The authors also found that treatment of the organoids with LRRK2 kinase inhibitors significantly reduced phosphorylated α-synuclein and restored expression of DA neuron markers [108]. Such findings are encouraging to stimulate similar work on patient-derived organoids.

In situ hybridization studies have reported similar neuroanatomical distributions of LRRK2 mRNA in mouse and rat brain [151]. Intriguingly, low levels of LRRK2 mRNA expression are reported in the SNpc, the region where neurodegeneration occurs in PD, whereas abundant LRRK2 expression is found in the projection region of the SNpc, the striatum. These findings of low LRRK2 expression in SNpc and high LRRK2 expression in striatum have been corroborated at the protein level by immunohistochemical LRRK2 detection in rodents [152,153], as well as in primates [154]. These observations prompted the hypothesis that LRRK2 effects on dopaminergic neurons in disease may be due in part to the effects in other cells. For instance, the substantia nigra is in contact with the striatum via mutual projections within the basal ganglia thalamocortical circuitry, and the nigral degeneration observed could be a consequence of abnormalities in this or related circuits. Within this circuit, LRRK2 is expressed in medium spiny neurons of the striatum that receive dopaminergic input and provide direct and indirect projections to the substantia nigra pars reticulata. LRRK2 is also expressed within cortical glutamatergic neurons that project to the striatal medium spiny neurons. In addition, we also know that LRRK2 is found in microglia that show low LRRK2 expression levels in basal conditions and a strong upregulation of LRRK2 when activated [155]. These expression patterns further support the notion that it is important to take into account LRRK2 functions in different cell types to complete the picture of correlations between LRRK2 phenotypes in patients in cell models.

From the descriptions of LRRK2 patients and LRRK2 patient-derived cells in the literature to date, what is most striking is that few markers used in patient phenotypes are common to markers used in patient-derived cells. This is of course not specific to LRRK2 but is a general consequence of the fact that patient cell analysis relies on biological markers while very few biological markers of disease are currently available in the clinic. Recently, many researchers have been looking into surrogate manifestations of disease in terms of a quantifiable biological marker, which can indicate disease prognosis or act as a means to monitor a therapeutic strategy. For instance, Fraser and colleagues reported an increase in phosphorylated S1292 (pS1292)–LRRK2 to total LRRK2 ratio in urinary exosomes of G2019S-LRRK2 mutation carrier [156]. A study by Wang and colleagues also showed an upregulation of pS1292 levels in exosomes obtained from the urine and cerebrospinal fluid (CSF) of G2019S–LRRK2 Norwegian subject cohort [157]. A recent study of Winter and colleagues also delves into incorporating urinary proteome profiling and machine learning to analyze and compare the biospecimens from LRRK2-associated PD patients and idiopathic patients. They reported a lysosomal dysregulation in urine samples collected from G2019S-LRRK2 pathogenic mutation carriers. Their study also suggested that the levels of neurotrophic factor VGF provided was a major determinant to distinguish between manifesting and non-manifesting disease [92]. Additional experimental work has been done using recently identified as LRRK2 substrates, Rab GTPases, as potential biomarkers of disease or therapeutic response [19]. For instance, phosphorylation of Rab10 is found to be increased compared to controls in neutrophils isolated from a small group of patients with idiopathic PD or PD with LRRK2-G2019S [158]. Rab phosphorylation has also been shown to be inhibited in neutrophils and PBMCs treated with LRRK2 kinase inhibitor [159,160]. These studies suggest the feasibility that biological readouts from patients could be developed and would open the opportunity to use the same readouts in patient-derived cell models. At the moment, few of these options to measure biological markers in patients has been translated into routine clinical practice. Therefore, enhancing the possibility to measure biological markers in patients is a necessary step to establish parallels between patients and patient-derived cells. Recent advances in LRRK2-related biomarkers have been reviewed by Rideout and colleagues [161].

Additional markers may be of interest to establish correlations between patients and cellular experiments. For instance, alterations in dopamine dynamics can be studied as dopamine transporter imaging is performed in PD patients [162]. It is also important to determine whether any kidney and lung phenotypes can be observed in patients with LRRK2 mutations owing to the fact that inhibition of LRRK2 kinase activity in non-human primates results in cytoplasmic accumulation of lamellar bodies in lung type II pneumatocytes or enlarged vacuoles in kidney cells [163]. Measures in gastrointestinal tract are another option, as it has been reported by Derkinderen and colleagues [164] through analysis of routine colonic biopsies that the α-synuclein pathology is present in almost all PD patients. Furthermore, the mutation LRRK2 N2081D which lies in the kinase domain of LRRK2, is a known risk factor of Crohn’s disease, it is interesting because PD patients often present with gastrointestinal disturbances such as constipation and dysphagia [165]. Finally, activation of inflammatory pathways has been hypothesized to trigger LRRK2 mutants to induce neurodegeneration that results in PD (reviewed in *Cabezudo* and colleagues [166]) and may offer additional opportunities for parallel measures in patients and patient-derived cells.

The goal of this review has been to draw parallels between what is observed in the clinical setting and what indicators we can look for in early stages to diagnose at risk PD patients, prior to it taking on a disabling and irreversible role in the patient’s life. While the relevance of this approach of comparing characteristics of patients with properties of patient-derived cells is comprehensible for carriers of LRRK2 coding sequence mutations, the work initiated must be pursued in order to establish the usefulness of such comparisons (see Box 1 of outstanding issues). Furthermore, thus far, little information is available on clinical patient phenotypes and patient cell-derived characteristics for non-coding variants that have been revealed by GWAS studies. It therefore remains to be verified if cells isolated from individuals carrying PD risk SNPs at the LRRK2 locus demonstrate phenotypes that can be put into relation with the disease. This ongoing analysis will allow us to begin to understand the links between what is observed in patients compared to what is observed in patient cells as well as to identify the gaps to be filled in order to establish strong correlations between the two.

Box 1 **Box of outstanding issues to close the gap in our understanding of how clinical patient phenotypes and cellular phenotypes of patient-derived cells.**

- In order to establish better and more quantitative correlations between clinical patient phenotypes and cellular phenotypes of patient-derived cells, there is a need to implement biological marker readouts that are analogous in patients and patient cells.
- Studies of clinical progression of disease are often performed on large cohorts of patients, while studies in patient-derived cell cultures are performed on average using a much lower numbers of patients. It is a challenge to determine what the optimal sample size is in studies with patient-derived cells, the task being to balance the need for high numbers to ensure the robustness of the findings with the limitations in throughput/capacity of research and clinical laboratories.
- Studies are accumulating with cells derived from subjects with coding sequence variants of LRRK2. However little attention has been given thus far to analyzing cells from subjects who carry risk factor variants that do not affect the LRRK2 coding sequence. As these may constitute low risk but more common variants, it would be important to also pursue studies in cells with non-coding risk factor variants of LRRK2.
- The usefulness of each patient-derived cell type in informing on the patient’s disease and/or response to therapy should be addressed. Similarly, situations can be identified where simple and quick protocols such as simple primary cultures relevant as opposed to other situations where more refined and sophisticated protocols are needed such as those involving cell de- and re-differentiation.
- In addition to studies using homogeneous cell cultures of patient-derived cells, more sophisticated culture configurations, such as 3D cultures, co-cultures and organoid cultures should be evaluated for their tractability to provide correlative information about patients’ disease state, treatment options and prognosis.
- In order to obtain PD specific information it is vital to have PD patients derived cells with non-LRRK2 mutations (mutations in other genes or idiopathic forms) as well as healthy controls to compare the baseline variation in the molecular mechanisms of reprogrammed cells among the three cohorts of cells derived. As PD is a progressive neurodegenerative disease it is vital to study the progressive changes in the molecular mechanisms of the patient-derived reprogrammed cells.
- An additional opportunity is to carry out studies on LRRK2 variants that are inversely correlated to risk for PD (the so-called ‘protective’ variants). Such studies have the potential to reveal molecular pathways that could be exploited as a strategy to offer neuroprotection to PD patients.

## Data Availability

No new data were created or analyzed in this study. Data sharing is not applicable to this article.
